# Blockade of TNF-α/TNFR2 signalling suppresses colorectal cancer and enhances the efficacy of anti-PD1 immunotherapy by decreasing CCR8^+^T regulatory cells

**DOI:** 10.1093/jmcb/mjad067

**Published:** 2023-11-03

**Authors:** Yixian Guo, Feng Xie, Xu Liu, Shouyu Ke, Jieqiong Chen, Yi Zhao, Ning Li, Zeyu Wang, Gang Yi, Yanying Shen, Dan Li, Chunchao Zhu, Zizhen Zhang, Gang Zhao, Hong Lu, Bin Li, Wenyi Zhao

**Affiliations:** Department of Gastrointestinal Surgery, Renji Hospital, Shanghai Jiao Tong University School of Medicine, Shanghai 200025, China; Shanghai Institute of Immunology, Shanghai Jiao Tong University School of Medicine, Shanghai 200025, China; Department of Immunology and Microbiology, Shanghai Jiao Tong University School of Medicine, Shanghai 200025, China; Department of Gastrointestinal Surgery, Renji Hospital, Shanghai Jiao Tong University School of Medicine, Shanghai 200025, China; Department of Gastrointestinal Surgery, Renji Hospital, Shanghai Jiao Tong University School of Medicine, Shanghai 200025, China; Shanghai Institute of Immunology, Shanghai Jiao Tong University School of Medicine, Shanghai 200025, China; Shanghai Institute of Immunology, Shanghai Jiao Tong University School of Medicine, Shanghai 200025, China; Department of Immunology and Microbiology, Shanghai Jiao Tong University School of Medicine, Shanghai 200025, China; Shanghai Institute of Immunology, Shanghai Jiao Tong University School of Medicine, Shanghai 200025, China; Department of Immunology and Microbiology, Shanghai Jiao Tong University School of Medicine Shanghai 200025, China; Department of Gastrointestinal Surgery, Renji Hospital, Shanghai Jiao Tong University School of Medicine, Shanghai 200025, China; Biotheus Inc., Zhuhai 519080, China; Department of Pathology, Renji Hospital, Shanghai Jiao Tong University School of Medicine, Shanghai 200025, China; Shanghai Institute of Immunology, Shanghai Jiao Tong University School of Medicine, Shanghai 200025, China; Department of Immunology and Microbiology, Shanghai Jiao Tong University School of Medicine, Shanghai 200025, China; Department of Gastrointestinal Surgery, Renji Hospital, Shanghai Jiao Tong University School of Medicine, Shanghai 200025, China; Department of Gastrointestinal Surgery, Renji Hospital, Shanghai Jiao Tong University School of Medicine, Shanghai 200025, China; Department of Gastrointestinal Surgery, Renji Hospital, Shanghai Jiao Tong University School of Medicine, Shanghai 200025, China; GI Division, Renji Hospital, Shanghai Jiao Tong University School of Medicine, Shanghai 200025, China; Shanghai Institute of Immunology, Shanghai Jiao Tong University School of Medicine, Shanghai 200025, China; Department of Immunology and Microbiology, Shanghai Jiao Tong University School of Medicine, Shanghai 200025, China; Department of Gastrointestinal Surgery, Renji Hospital, Shanghai Jiao Tong University School of Medicine, Shanghai 200025, China

**Keywords:** TNFR2, CCR8, regulatory T cells, colorectal cancer

## Abstract

The enrichment of regulatory T cells (Tregs) in the tumour microenvironment (TME) has been recognized as one of the major factors in the initiation and development of resistance to immune checkpoint inhibitors. C–C motif chemokine receptor 8 (CCR8), a marker of activated suppressive Tregs, has a significant impact on the functions of Tregs in the TME. However, the regulatory mechanism of CCR8 in Tregs remains unclear. Here, we revealed that a high level of TNF-α in the colorectal cancer (CRC) microenvironment upregulated CCR8 expression in Tregs via the TNFR2/NF-κB signalling pathway and the FOXP3 transcription factor. Furthermore, in both anti-programmed cell death protein 1 (anti-PD1)-responsive and anti-PD1-unresponsive tumour models, PD1 blockade induced CCR8^+^ Treg infiltration. In both models, *Tnfr2* depletion or TNFR2 blockade suppressed tumour progression by reducing CCR8^+^ Treg infiltration and thus augmented the efficacy of anti-PD1 therapy. Finally, we identified that TNFR2^+^CCR8^+^ Tregs but not total Tregs were positively correlated with adverse prognosis in patients with CRC and gastric cancer. Our work reveals the regulatory mechanisms of CCR8 in Tregs and identifies TNFR2 as a promising target for immunotherapy.

## Introduction

Immune checkpoint inhibitors (ICIs), which are monoclonal antibodies (mAbs) against programmed cell death protein 1 (PD1), have revolutionized the field of cancer immunotherapy (Ganesh et al., 2019). However, treatment resistance is still a major concern, especially for patients with colorectal cancer (CRC) (Oliveira et al., 2019). CRC can be classified into two molecular subtypes: microsatellite stable (MSS) and microsatellite instable (MSI) (Jass, 2007). Only patients in the MSI subtype respond to ICI therapy, while patients in the MSS subtype, accounting for 85% of total CRC, have poor therapeutic response (Dudley et al., 2016). The complex immune microenvironment is one of the reasons for the initiation and development of resistance to ICI therapy, and regulatory T cells (Tregs) may play crucial roles in this process (Facciabene et al., 2012; Sharma et al., 2017).

Tregs, an immunosuppressive subset of CD4^+^ T cells, are components of the immune system and play essential roles in maintaining self-tolerance. The roles of Tregs in CRC remain controversial (Salama et al., 2009; De Simone et al., 2016; Waniczek et al., 2017). The heterogeneity of Treg subsets, which are phenotypically similar but functionally divergent, may explain these conflicting findings (Picard et al., 2020). Obviously, it is essential to identify the activated suppressive Treg subsets in the tumour microenvironment (TME).

Many studies have found that C–C motif chemokine receptor 8 (CCR8), a member of the β-chemokine receptor family, is a marker of activated suppressive Tregs in the TME. CCR8 signalling contributes to the suppressive function and survival of donor Tregs in a murine model of graft-versus-host disease (Coghill et al., 2013). Moreover, CCR8 expression in Tregs plays a pivotal role in the progression of breast cancer (Plitas et al., 2016). Our previous study showed that CCR8^+^ Tregs were highly infiltrative and had a stronger immunosuppressive ability than CCR8^−^ Tregs in pancreatic cancer (Yi et al., 2018). However, the precise functions of CCR8^+^ Tregs and the molecular mechanism underlying the upregulation of CCR8 expression in CRC Tregs are still unclear.

The activation and regulation of Tregs are orchestrated by inflammatory factors (Rochman et al., 2009; Toomer and Malek, 2018). TNF-α is a pleiotropic cytokine that has proinflammatory and immunosuppressive effects, depending on the context, duration of exposure, and disease state (Wang and Lin, 2008). In the TME, TNF-α exhibits both pro- and antitumoural effects, which are transmitted through the TNFR1 and TNFR2 receptors (Ham et al., 2016). The TNF-α/TNFR2 interaction increases the survival, recruitment, and expansion of Tregs, which suppresses antitumour immunity in mouse cancer models (Chen and Oppenheim, 2010; Chang et al., 2015). Additionally, pretreatment of Tregs with TNF-α increases their capacity to suppress antitumour immunity after adoptive transfer (Sasi et al., 2012). However, only a few studies have investigated the effects of TNF-α/TNFR2 on Tregs in the TME. Here, we reported that in both MC38 cell-based and CT26 cell-based tumour models (Limagne et al., 2019), anti-PD1 treatment induced CCR8^+^ Treg infiltration, thus promoting immune escape. Moreover, the depletion of *Tnfr2* or blockade of TNFR2 inhibited tumour growth and enhanced the therapeutic efficacy of the anti-PD1 mAb by reducing CCR8^+^ Treg invasion. In addition, TNFR2^+^CCR8^+^ Tregs, but not total Tregs, in the TME were positively correlated with clinicopathological characteristics and prognosis in CRC patients. Our study highlights the key role of TNF-α/TNFR2 in the regulation of CCR8^+^ Tregs in the antitumour immune response.

## Results

### TNF-α upregulates CCR8 expression in Tregs in the CRC microenvironment

**Figure 1:**
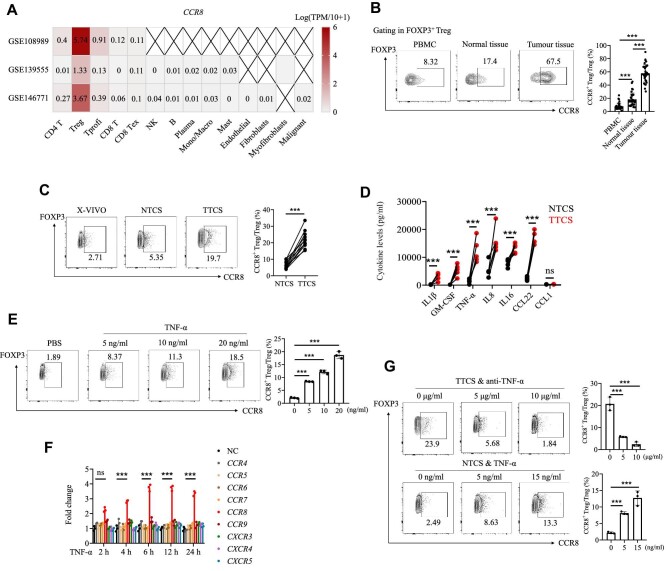
TNF-α from the TME induces the CCR8^+^ Treg subset. (**A**) *CCR8* expression in various cell types from different CRC single-cell sequencing datasets (Sun et al., 2021). (**B**) PBMCs, normal tissue samples, and tumour tissue samples were collected from CRC patients undergoing surgery at Renji Hospital. The percentage of CCR8^+^ Tregs in PBMCs, normal tissues, and tumour tissues was measured by FACS. (**C**) Tregs derived from healthy donors were cultured with autologous TTCS and NTCS for 48 h. Then, the percentage of CCR8^+^ Tregs was measured by FACS. (**D**) Luminex multifactor detection of variations in cytokine secretion between TTCS and NTCS samples derived from CRC patients. (**E**) Tregs derived from healthy donors were treated with TNF-α (5, 10, and 20 ng/ml) or PBS for 48 h. Then, the percentages of CCR8^+^ Tregs were measured by FACS. (**F**) Tregs derived from healthy donors were treated with TNF-α (10 ng/ml) for 2, 4, 6, 12, and 24 h. Quantitative real-time PCR analysis of *CCR4, CCR6, CCR7, CCR8, CCR9, CXCR3, CXCR4*, and *CXCR5* expression in Tregs. (**G**) Tregs derived from healthy donors were cultured with TTCS plus anti-TNF-α mAb (5 and 10 μg/ml) or NTCS plus TNF-α (5 and 15 ng/ml) for 48 h. Then, the percentage of CCR8^+^ Tregs was measured by FACS. The data are representative of three independent experiments. All the data are presented as mean ± SD. ***P < 0.001; ns, not significant.

According to our previous study, CCR8 was specifically expressed in activated tumour-infiltrating suppressive Tregs in pancreatic cancer (Yi et al., 2018). By analysing different single-cell sequencing data for CRC (Sun et al., 2021), we found that CCR8 was also specifically expressed in tumour-infiltrating Tregs in CRC (Figure 1A). The analysis of fresh CRC samples indicated that CCR8^+^ Tregs were highly infiltrative in the CRC microenvironment (Figure 1B). To further explore the underlying mechanisms, we isolated Tregs from peripheral blood mononuclear cells (PBMCs) of treatment-naïve CRC patients and cultured them with tumour tissue culture supernatant (TTCS) or normal tissue culture supernatant (NTCS) obtained from fresh tumour tissues and corresponding cancer-adjacent tissues, respectively. After 48 h of culture, CCR8 expression was induced by TTCS but not by NTCS (Figure 1C). To explore which cytokines in the TTCS were involved in this process, we used Luminex multifactor detection to identify cytokines present in the TTCS. Out of 30 cytokines tested, the levels of six cytokines were higher in TTCS than in NTCS (Figure 1D). Tregs were then stimulated with these six cytokines. Only TNF-α enhanced CCR8 expression in Tregs at both the translational and transcriptional levels (Figure 1E and F). However, TNF-α was unable to induce the expression of other members of the CCR family (Figure 1F). Furthermore, the addition of an anti-TNF-α mAb to the TTCS inhibited the induction of CCR8^+^ Tregs, and the addition of TNF-α to the NTCS induced CCR8^+^ Tregs (Figure 1G). Moreover, after pretreatment with TNF-α for 48 h, Tregs displayed higher expression levels of suppressive markers (Supplementary Figure S1A). TNF-α-treated Tregs also significantly suppressed CD8^+^ T cell proliferation (Supplementary Figure S1B) and the secretion of effector cytokines (Supplementary Figure S1C) in CD8^+^ T cells. These data indicated that TNF-α might be key to inducing the transformation of CCR8^−^ Tregs into CCR8^+^ Tregs in the TME.

### TNF-α upregulates CCR8 expression in Tregs via the TNFR2/NF-κB pathway and the FOXP3 protein

**Figure 2:**
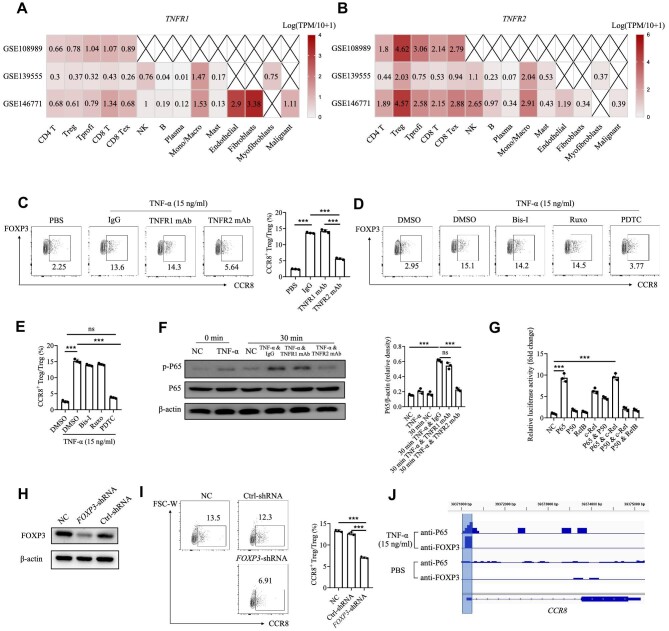
TNF-α upregulates CCR8 expression in Tregs via the TNFR2/NF-κB signalling pathway and the FOXP3 protein. (**A**) *TNFR1* expression in various cell types from different CRC single-cell sequencing datasets (Sun et al., 2021). (**B**) *TNFR2* expression in various cell types from different CRC single-cell sequencing datasets (Sun et al., 2021). (**C**) Tregs derived from healthy donors were treated with TNF-α (15 ng/ml) combined with the anti-TNFR1 mAb (5 μg/ml), anti-TNFR2 mAb (5 μg/ml), or the IgG isotype control. Then, the percentage of CCR8^+^ Tregs was measured by FACS. (**D** and **E**) Tregs derived from healthy donors were treated with TNF-α (15 ng/ml) followed by the addition of various inhibitors of different signalling pathways, including bisindolylmaleimide I (Bis-I; a PKC inhibitor, 27 nM), ruxolitinib (Ruxo; a STAT3 inhibitor, 3.3 nM), and PDTC (an NF-κB inhibitor, 20 μM). Then, the percentage of CCR8^+^ Tregs was measured by FACS. (**F**) Tregs derived from healthy donors were treated with TNF-α or TNF-α plus anti-TNFR1 mAb or TNF-α plus anti-TNFR2 mAb. The protein levels of P65 and p-P65 were measured by western blotting. NC, negative control. (**G**) The binding ability of NF-κB subunits to the CCR8 promoter region was determined by the luciferase reporter assay. (**H**) Tregs derived from healthy donors were transfected with *FOXP3*-shRNA or negative control-shRNA. FOXP3 protein levels were measured by western blotting. (**I**) Tregs transfected with *FOXP3*-shRNA or negative control-shRNA were stimulated with TNF-α (15 ng/ml). Then, the percentage of CCR8^+^ Tregs was measured by FACS. (**J**) CUT&RUN identification of P65 and FOXP3 binding sites in the promoter region of CCR8. The data are representative of three independent experiments. All the data are presented as mean ± SD. ***P < 0.001; ns, not significant.

TNF-α has two main receptors, TNFR1 and TNFR2 (Sun et al., 2021). To determine which TNF-α receptor is mainly responsible for the regulation of CCR8, we analysed single-cell sequencing data. The results showed that TNFR2, rather than TNFR1, was highly expressed in FOXP3^+^ Tregs, indicating that the effect of TNF-α on Tregs might be TNFR2-dependent (Figure 2A and B).

The activation of inflammation-associated signalling pathways has been implicated in the regulation of T cell functions. We used an anti-TNFR1 mAb, an anti-TNFR2 mAb, and an isotype control to investigate the possible mechanisms by which TNF-α upregulates the expression of CCR8. We found that the blockade of TNFR2 by the anti-TNFR2 mAb inhibited TNF-α-induced CCR8 expression in Tregs at both the mRNA and protein levels (Figure 2C; Supplementary Figure S2A). In addition, TNF-α induced CCR8 expression in Tregs from wild-type (WT) mice but not in Tregs from *Tnfr2* knockout (*Tnfr2*^−/−^) mice (Supplementary Figure S2B). To assess which signalling pathway regulates CCR8 expression in Tregs, small molecule inhibitors targeting specific pathways were used, including ruxolitinib, which blocks the STAT3 signalling pathway, pyrrolidine dithiocarbamate (PDTC), which blocks the NF-κB signalling pathway, and bisindolylmaleimide I, which blocks the protein kinase C (PKC) signalling pathway (Figure 2D and E). We found that the NF-κB signalling pathway inhibitor PDTC attenuated TNF-α-induced CCR8 expression. Western blot analysis further confirmed that TNF-α upregulated the expression of CCR8 in Tregs via the TNFR2/NF-κB signalling pathway (Figure 2F). Next, we performed luciferase assays to determine the subunits of NF-κB that were involved in this process. Our results confirmed that P65 exhibited the most significant effect on upregulating CCR8 expression (Figure 2G).

We also noted that among all T cells, TNF-α only upregulated CCR8 expression in FOXP3^+^ Tregs, suggesting that FOXP3 might play an essential role in this process (Supplementary Figure S3). A short-hairpin ribonucleic acid (shRNA) lentivirus was designed to knock down *FOXP3* expression in Tregs isolated from the peripheral blood of healthy donors (Figure 2H). Compared with the controls, Tregs in the *FOXP3*-knockdown group had the lowest increase in CCR8 expression (Figure 2I), indicating that FOXP3 might be involved in the TNF-α-mediated upregulation of CCR8 expression in Tregs. Using the CUT&RUN assay and sequencing of the enriched DNA, we detected the binding of FOXP3 and P65 to the *CCR8* promoter region in Tregs after TNF-α stimulation, which promoted the transcriptional regulation and expression of CCR8 in Tregs (Figure 2J). These results highlighted the potential role of FOXP3 and P65 in maintaining CCR8 expression, which was necessary to establish this specific tumour-infiltrating Treg subset.

### TNFR2^+^CCR8^+^ Tregs are highly infiltrative in CRC and show a strong immunosuppressive capability

**Figure 3:**
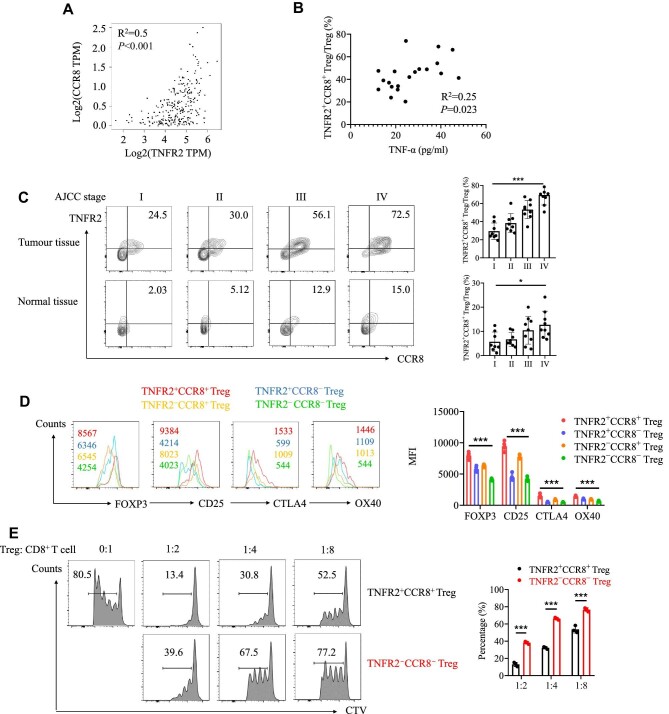
TNFR2^+^CCR8^+^ Tregs are highly infiltrative in CRC and show a greater immunosuppressive capability. (**A**) The correlation between TNFR2 and CCR8 in CRC was analysed by GEPIA2, an online TCGA database (Jenkins et al., 2018). TPM, transcripts per million. R^2^, coefficient of determination. (**B**) Analysis of the correlation between TNF-α and TNFR2^+^CCR8^+^ Tregs in tumour tissues from CRC patients (*n* = 20). (**C**) FACS analysis of the histologic grade-based percentages of TNFR2^+^CCR8^+^ Tregs in tumour and normal tissues (grade I, *n* = 8; grade II, *n* = 8; grade III, *n* = 9; and grade IV, *n* = 9). AJCC, American Joint Committee on Cancer. (**D**) Tumour-infiltrating Tregs were isolated from CRC patient samples. Functional markers (FOXP3, CD25, CTLA4, and OX40) in TNFR2^+^CCR8^+^ Tregs, TNFR2^+^CCR8^−^ Tregs, TNFR2^−^CCR8^+^ Tregs, and TNFR2^−^CCR8^−^ Tregs were measured by FACS. MFI, mean fluorescence intensity. (**E**) Tumour-infiltrating TNFR2^+^CCR8^+^ Tregs or TNFR2^−^CCR8^+^ Tregs were isolated from CRC patient samples by FACS. CD8^+^ T cells were isolated from healthy donors by magnetic beads. CellTrace Violet (CTV)-labelled CD8^+^ T cells and Tregs were cocultured for 72 h at different ratios (0:1, 1:2, 1:4, and 1:8), and then the proliferative ability of CD8^+^ T cells was measured by FACS. All the data are represented as mean ± SD. *P < 0.05; ***P < 0.001; ns, not significant.

Based on the above findings, TNFR2 has been proposed as the core molecule involved in the TNF-α-mediated induction of CCR8 in Tregs. Next, we explored the relationship between CCR8 and TNFR2 in CRC using fresh patient samples. Analysis of data from The Cancer Genome Atlas (TCGA) database confirmed the positive correlation between CCR8 and TNFR2 in CRC (Figure 3A). Immunofluorescence staining of tumour tissue showed that TNFR2 and CCR8 were coexpressed in FOXP3^+^ Tregs (Figure 8A). Tumour-infiltrating Tregs from 34 CRC patients were sorted and analysed by fluorescence-activated cell sorting (FACS). Standard gating strategies were used to identify FOXP3^+^ Tregs (Supplementary Figure S4A). We not only found that TNFR2 and CCR8 were coexpressed in Tregs but also found that the increase in the number of TNFR2^+^CCR8^+^ Tregs was associated with tumour stage progression (Figure 3C). In addition, in tumour tissues, the concentration of TNF-α was positively correlated with the number of TNFR2^+^CCR8^+^ Tregs (Figure 3B).

Different Treg subsets might play distinct roles in tumours. To investigate the function of TNFR2^+^CCR8^+^ Tregs in the TME, we analysed the phenotypes and functions of tumour-infiltrating Tregs by FACS. The results demonstrated that the protein expression levels of CD25, FOXP3, CTLA4, and OX40 were considerably higher in TNFR2^+^CCR8^+^ Tregs than in other subtypes (Figure 3D). Next, we isolated tumour-infiltrating TNFR2^+^CCR8^+^ Tregs and TNFR2^−^CCR8^−^ Tregs from tumour tissues and found that TNFR2^+^CCR8^+^ Tregs, rather than TNFR2^−^CCR8^−^ Tregs, strongly suppressed the proliferation of CD8^+^ T cells (Figure 3E).

**Figure 4:**
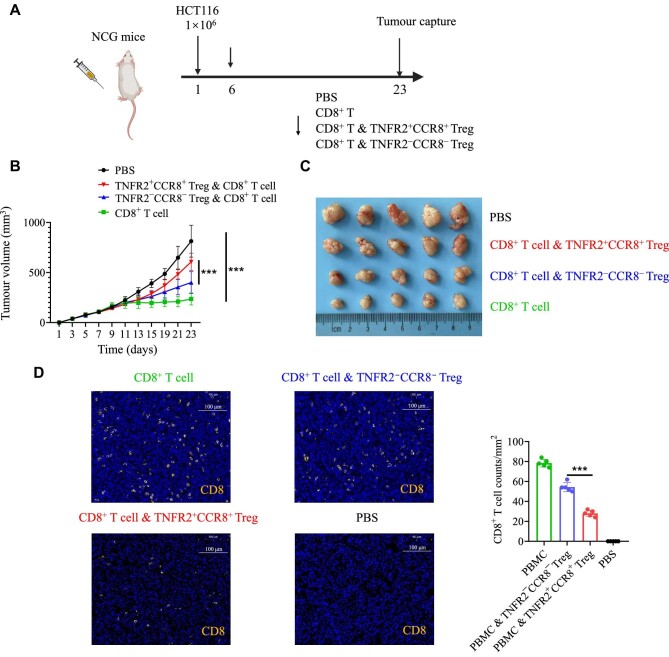
TNFR2^+^CCR8^+^ Tregs show stronger immunosuppression than TNFR2^−^CCR8^−^ Tregs *in vivo*. (**A**) NCG mice were injected with human HCT116 cells. Five days after tumour implantation, tumour-specific immune cells or the PBS control was injected into these mice. On Day 23, the mice were sacrificed. (**B**) Tumour volumes were measured every two days. The plot shows the tumour growth curve. (**C**) Tumour volumes on Day 23. (**D**) The counts of tumour-infiltrating CD8^+^ T cells in different groups were measured by immunofluorescence staining. The data are representative of three independent experiments. All the data are presented as mean ± SD. ***P < 0.001.

Furthermore, we established a cell line-derived xenograft (CDX) model by subcutaneous injection of HCT116 cells into NCG mice. Tumour-specific immune cells (CD8^+^ T cells, CD8^+^ T cells plus TNFR2^+^CCR8^+^ Tregs, or CD8^+^ T cells plus TNFR2^−^CCR8^−^ Tregs) or the phosphate-buffered saline (PBS) control was injected into the CDX model five days after tumour inoculation (Figure 4A). As expected, the injection of CD8^+^ T cells significantly suppressed tumour growth, whereas the injection of CD8^+^ T cells plus TNFR2^+^CCR8^+^ Tregs had no inhibitory effect on tumour growth progression (Figure 4B and C). The immunofluorescence staining results revealed more infiltrated CD8^+^ T cells in the TNFR2^−^CCR8^−^ Treg group than in the TNFR2^+^CCR8^+^ Treg group (Figure 4D). Taken together, the above *in vivo* and *in vitro* results confirmed that TNFR2^+^CCR8^+^ Tregs played crucial roles in inhibiting CD8^+^ T cell function and infiltration in the TME, thereby promoting tumour growth.

### CCR8^+^ Tregs are more easily retained in the TME by CCL18^+^ macrophages

**Figure 5:**
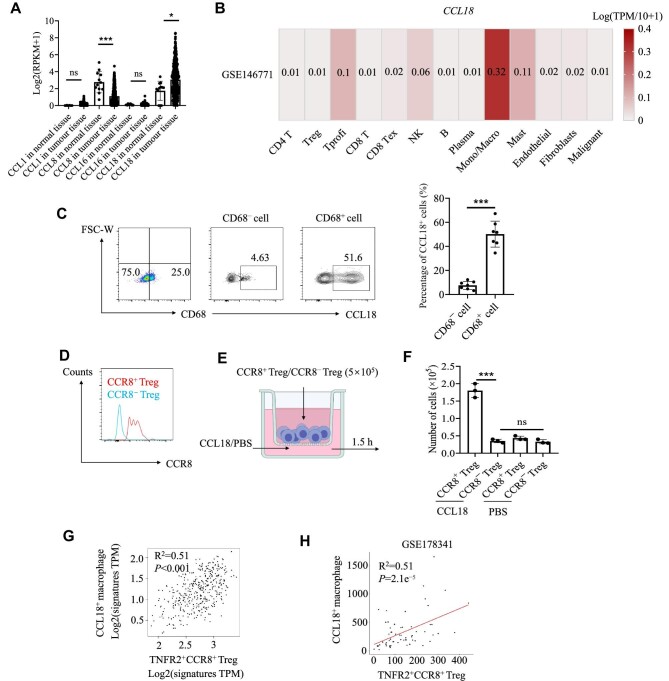
CCR8^+^ Tregs are more easily retained by CCL18^+^ macrophages in the TME than CCR8^−^ Tregs. (**A**) The expression levels of the CCR8 ligands CCL1, CCL8, CCL16, and CCL18 in CRC samples from the TCGA database. (**B**) Expression levels of CCL18 across different cell types from patients with CRC (Sun et al., 2021). (**C**) Tumour-infiltrating macrophages were isolated from CRC patient samples. The percentages of CCL18^+^CD68^+^ cells and CCL18^+^CD68^−^ cells were measured by FACS. (**D**) Tregs were obtained from the blood of healthy donors. The expression of CCR8 in CCR8^+^ Tregs and CCR8^−^ Tregs was measured by FACS. (**E**) CCR8^+^ Tregs and CCR8^−^ Tregs were isolated by FACS. Tregs (5 × 10^5^) were placed in the top chamber, and CCL18 was added to the bottom chamber. The chambers were incubated for 1 h, and the number of migrated Tregs in the bottom chamber was counted under a microscope. (**F**) The number of Tregs that migrated into the bottom chamber. (**G**) The correlation between CCL18^+^ macrophages and CCR8^+^ Tregs in CRC was analysed by GEPIA2, an online TCGA database (Tang et al., 2019). (**H**) The correlation between CCL18^+^ macrophages and CCR8^+^ Tregs across 62 CRC patients in the GSE178341 dataset (Pelka et al., 2021). The data are representative of three independent experiments. All the data are presented as mean ± SD. *P < 0.05; ***P < 0.001; ns, not significant.

To further elucidate the function of CCR8 in Tregs, we analysed the expression of CCR8 ligands in patients with CRC using datasets from the TCGA database. We noted that only CCL18 expression was significantly upregulated in tumour tissues compared with normal tissues (Figure 5A). Single-cell sequencing data revealed that CCL18 was mainly expressed in macrophages within the TME (Figure 5B; Sun et al., 2021). Flow cytometry analysis of fresh tumour samples verified that CCL18 was specifically expressed in CD68^+^ macrophages in the CRC microenvironment (Figure 5C). We then sorted CCR8^+^ Tregs induced by TNF-α stimulation and CCR8^−^ Tregs by FACS (Figure 5D) and performed a T cell transwell experiment (Figure 5E). The results showed that CCL18 was able to attract CCR8^+^ Tregs instead of CCR8^−^ Tregs (Figure 5F). Furthermore, analysis of TCGA data indicated that CCR8^+^ Tregs were positively associated with CCL18^+^ macrophages in the TME (Figure 5G). RNA-seq data from 62 CRC patients also showed a significant positive correlation between the infiltration of CCL18^+^ macrophages and TNFR2^+^ Tregs (Figure 5H). All of these results suggested that tumour-infiltrating CCR8^+^ Tregs were retained in the TME by CCL18^+^ macrophages.

### *Tnfr2* depletion reduces CCR8^+^ Treg infiltration in the CRC microenvironment and enhances the therapeutic efficacy of anti-PD1 therapy

**Figure 6:**
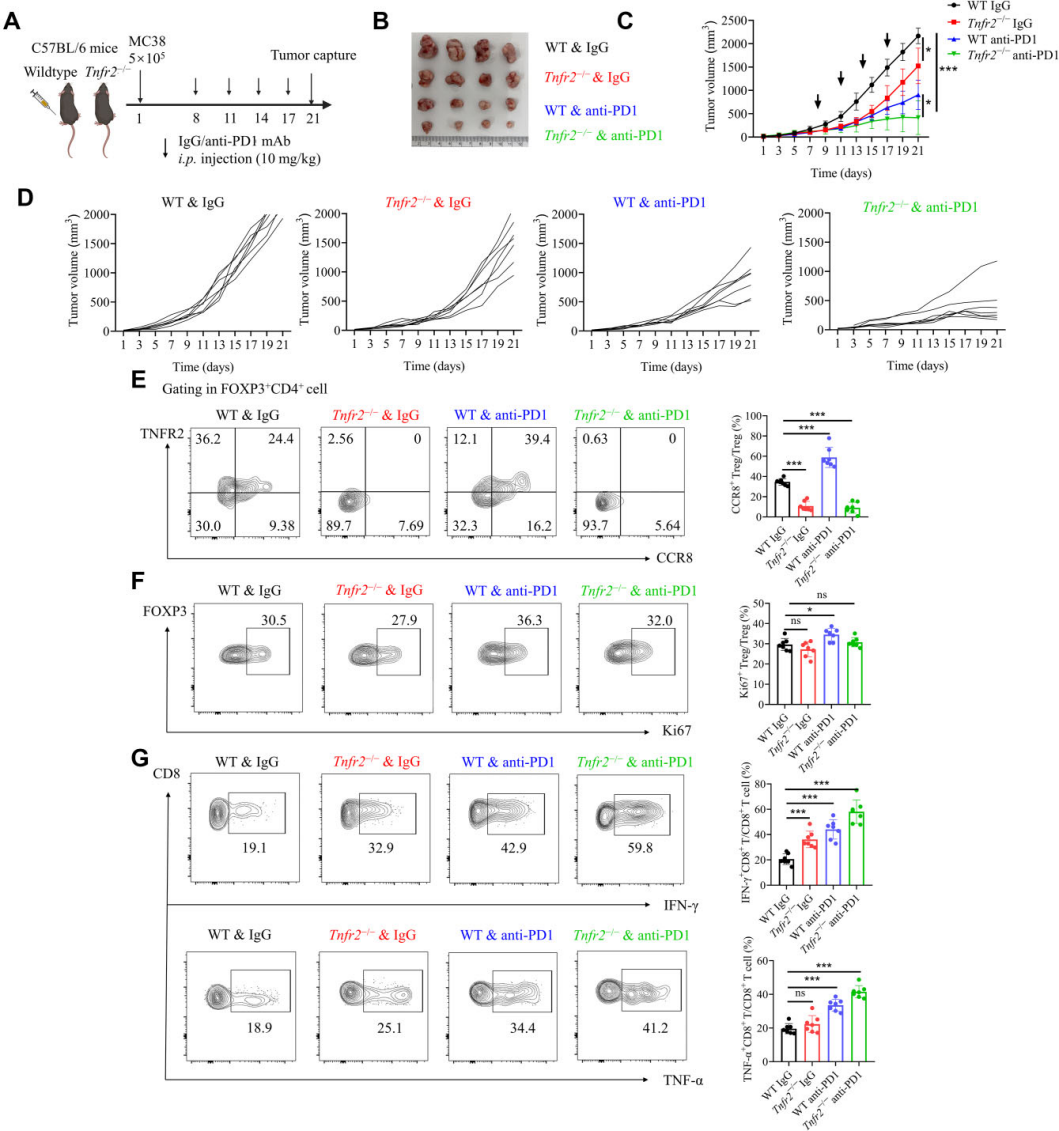
*Tnfr2* depletion inhibits tumour growth by reducing CCR8^+^ Treg infiltration and reversing anti-PD1 mAb-induced CCR8^+^ Treg infiltration. (**A**) WT and *Tnfr2^−^^/^^−^* C57BL/6 mice were inoculated with 5 × 10^5^ MC38 cells. Seven days after tumour inoculation, the mice received anti-PD1 mAb or IgG isotype control treatment. (**B**) On Day 21, the mice were sacrificed and tumour volumes were measured. (**C**) Tumour volumes were measured every two days. The plot shows the tumour growth curve. (**D**) Tumour growth curve for each mouse. (**E**) FACS and statistical analysis of CCR8^+^ Tregs from xenografts. (**F**) FACS and statistical analysis of Ki67^+^ Tregs from xenografts. (**G**) FACS and statistical analysis of TNF-α^+^CD8^+^ and IFN-γ^+^CD8^+^ T cells from xenografts. The data are representative of three independent experiments. All the data are presented as mean ± SD. *P < 0.05; ***P < 0.001; ns, not significant.

To further verify the conclusion that TNFR2 acts as the core molecule involved in CCR8^+^ Treg infiltration in CRC, we constructed *Tnfr2^−^^/^^−^* mice. The MC38 tumour cell line was used to establish subcutaneous CRC models in *Tnfr2^−^^/^^−^* and WT mice (Figure 6A). Tumour growth in the *Tnfr2^−^^/^^−^* group was notably slower than that in the WT group (Figure 6B–D). Flow cytometry analysis showed a significantly decreased percentage of CCR8^+^ Tregs in total Tregs (Figure 6E) in *Tnfr2^−^^/^^−^* mice, while the percentage of Ki67^+^ Tregs in total Tregs was not changed (Figure 6F). Furthermore, the expression levels of interferon-γ (IFN-γ) and TNF-α in CD8^+^ T cells were markedly increased (Figure 6G). The above results suggested that the depletion of *Tnfr2* could limit tumour growth by altering Treg subtypes, instead of inhibiting Treg proliferation, within the TME.

Interestingly, the *Tnfr2^−^^/^^−^* mice exhibited better therapeutic efficacy after anti-PD1 mAb treatment (Figure 6B–D). The proportion of CCR8^+^ Tregs increased significantly in the anti-PD1 mAb-treated WT group, but not in the anti-PD1 mAb-treated *Tnfr2^−^^/^^−^* group (Figure 6E), indicating that anti-PD1 therapy might induce CCR8^+^ Treg infiltration in the TME and that *Tnfr2* depletion could reverse this process. In addition, the CD8^+^ T cells in *Tnfr2^−^^/^^−^* mice treated with an anti-PD1 mAb had significantly higher levels of IFN-γ and TNF-α (Figure 6G). The above results suggested that PD1 blockade induced CCR8^+^ Treg infiltration, which might inhibit the antitumour functions of CD8^+^ T cells in the TME, resulting in CRC resistance to anti-PD1 therapy. Notably, the depletion of *Tnfr2* inhibited CCR8^+^ Treg infiltration in the TME. Therefore, the combination of *Tnfr2* depletion and PD1 blockade enhanced the therapeutic efficacy of anti-PD1 therapy.

### Anti-TNFR2 mAb treatment inhibits tumour growth and improves the curative effect of anti-PD1 therapy by reducing TNFR2^+^CCR8^+^ Treg infiltration

**Figure 7:**
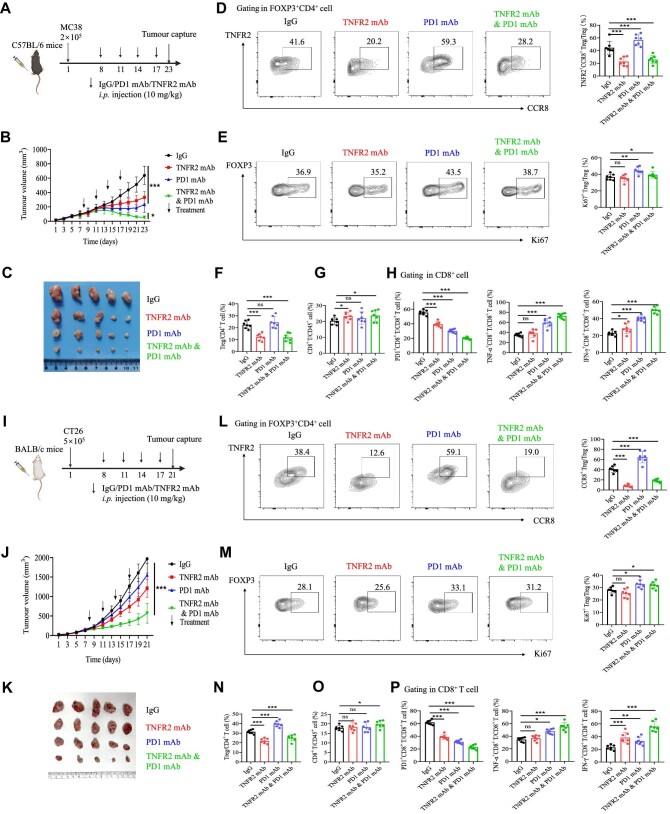
TNFR2 blockade inhibits tumour growth and improves the curative effect of anti-PD1 treatment by reducing CCR8^+^ Treg infiltration. (**A**) WT C57BL/6 mice were injected with 2 × 10^5^ MC38 cells. Mice received anti-PD1 mAb, anti-TNFR2 mAb, or IgG isotype control treatment seven days after tumour inoculation. On Day 23, the mice were sacrificed. (**B**) Tumour volumes were measured every two days. The plot shows the tumour growth curve. (**C**) Tumour volumes on Day 23. (**D**) FACS and statistical analysis of TNFR2^+^CCR8^+^ Tregs from MC38 tumour xenografts. (**E**) FACS and statistical analysis of Ki67^+^ Tregs from MC38 tumour xenografts. (**F**) Percentages of Tregs in CD4^+^ T cells from MC38 tumour xenografts. (**G**) The ratios of CD8^+^ T cells from MC38 tumour xenografts. (**H**) Percentages of PD1^+^CD8^+^, TNF-α^+^CD8^+^, and IFN-γ^+^CD8^+^ T cells in CD8^+^ T cells from MC38 tumour xenografts. (**I**) WT BALB/c mice were injected with 5 × 10^5^ CT26 cells. Seven days after tumour inoculation, the mice received anti-PD1 mAb, anti-TNFR2 mAb, or IgG isotype control treatment. On Day 21, the mice were sacrificed. (**J**) Tumour volumes were measured every two days. The plot shows the tumour growth curve. (**K**) Tumour volumes on Day 21. (**L**) FACS and statistical analysis of TNFR2^+^CCR8^+^ Tregs from CT26 tumour xenografts. (**M**) FACS and statistical analysis of Ki67^+^ Tregs from CT26 tumour xenografts. (**N**) Percentages of Tregs in CD4^+^ T cells from CT26 tumour xenografts. (**O**) The ratios of CD8^+^ T cells from CT26 tumour xenografts. (**P**) Percentages of PD1^+^CD8^+^, TNF-α^+^CD8^+^, and IFN-γ^+^CD8^+^ T cells in CD8^+^ T cells from CT26 tumour xenografts. The data are representative of three independent experiments. All the data are presented as mean ± SD. *P < 0.05; **P < 0.01; ***P < 0.001; ns, not significant.

To further clarify the therapeutic potential of targeting TNFR2 in CRC treatment, we used MC38 cells and CT26 cells to establish murine subcutaneous tumour models. One week after MC38 tumour cell inoculation, the mice were treated with the anti-PD1 mAb, anti-TNFR2 mAb, anti-PD1 plus anti-TNFR2 mAbs, or the immunoglobulin G (IgG) isotype control (Figure 7A). Anti-TNFR2 mAb treatment alone appreciably limited tumour growth compared to the IgG isotype control. The most significant tumour growth inhibition was observed in the group treated with both anti-PD1 and anti-TNFR2 mAbs (Figure 7B and C). Next, we assessed the number of CD8^+^ T cells and Tregs in the TME and found that there was no difference in the proportion of CD8^+^ T cells among the treatment groups, but the differences in the Treg proportion were statistically significant among the treatment groups (Figure 7F and G). More precisely, TNFR2^+^CCR8^+^ Tregs decreased sharply in the anti-TNFR2 mAb-treated groups, while they increased significantly in the group that only received the anti-PD1 mAb (Figure 7D). There was no significant difference in the proportion of Ki67^+^ Tregs between the IgG- and anti-TNFR2 mAb-treated groups, indicating that Tregs in the TME might have a similar proliferative capacity (Figure 7E). Compared with the control, TNFR2 blockade significantly increased the infiltration of IFN-γ^+^CD8^+^ T cells while decreasing the infiltration of PD1^+^CD8^+^ T cells in the TME. The combination of anti-PD1 and anti-TNFR2 treatment showed a significantly higher percentage of cytotoxic CD8^+^ T cell (IFN-γ^+^CD8^+^ or TNF-α^+^CD8^+^ T cells) infiltration in the TME than any monotherapy (Figure 7H).

Similar to MC38 cells, CT26 cells were inoculated into BALB/c mice, and the mice were treated with anti-PD1 mAb and/or anti-TNFR2 mAb (Figure 7I). In comparison with the MC38 model, anti-TNFR2 but not anti-PD1 treatment showed a better tumour inhibitory effect, indicating that CT26 mouse tumours might not respond to anti-PD1 treatment. However, the combination of both mAbs significantly improved the therapeutic effect of single mAb treatment (Figure 7J and K). Next, tumour-infiltrating Tregs and CD8^+^ T cells were assessed by flow cytometry. The proportions of CD8^+^ T cells were similar among the different treatment groups, while the proportions of Tregs varied greatly (Figure 7N and O). The percentage of CCR8^+^ Tregs decreased markedly in the anti-TNFR2 treatment groups but increased significantly in the group that received only the anti-PD1 mAb (Figure 7L). The proportions of Ki67^+^ Tregs were similar among the different treatment groups (Figure 7M). More importantly, the combination of anti-PD1 and anti-TNFR2 treatment showed a significantly higher percentage of cytotoxic CD8^+^ T cell (IFN-γ^+^CD8^+^ or TNF-α^+^CD8^+^ T cells) infiltration in the TME than any monotherapy (Figure 7P).

Taken together, these results suggested that, regardless of whether the tumour models responded to PD1 blockade, TNFR2 blockade could suppress tumour growth and enhance the therapeutic effect of anti-PD1 treatment by boosting the functions of cytotoxic CD8^+^ T cells and reducing the infiltration, not proliferation, of CCR8^+^ Tregs.

### TNFR2^+^CCR8^+^ Tregs are negatively correlated with the overall survival of CRC and gastric cancer patients

**Figure 8:**
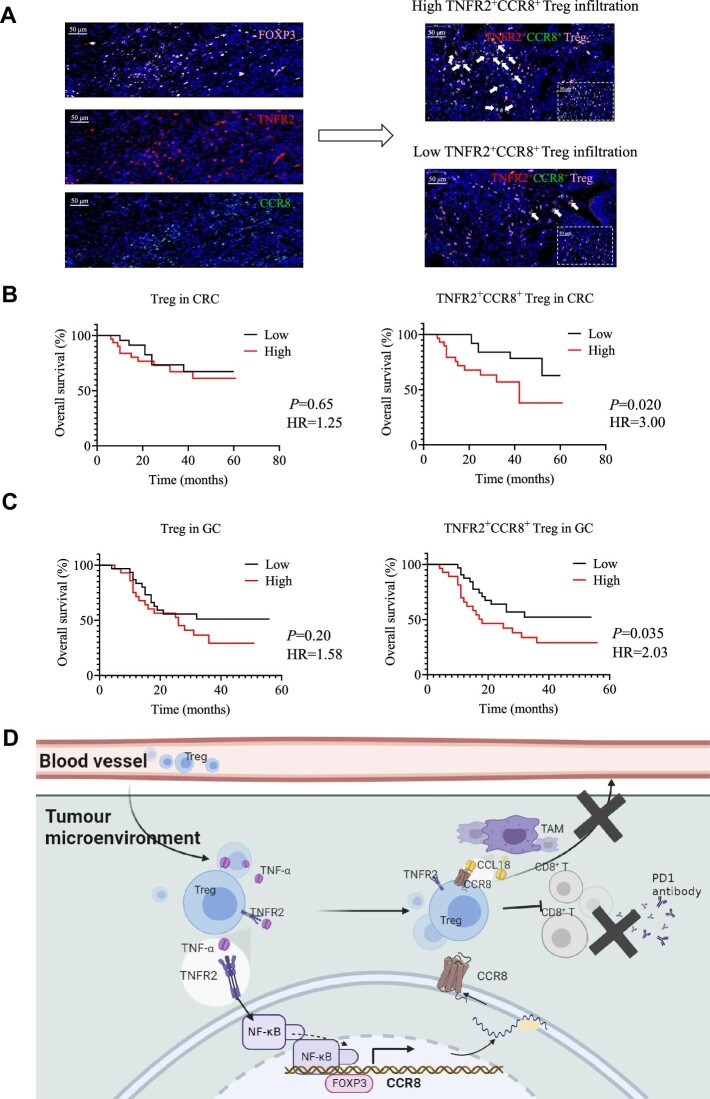
A high level of TNFR2^+^CCR8^+^ Tregs is significantly associated with poor overall survival of CRC and GC patients. (**A**) Immunofluorescence staining of TNFR2^+^CCR8^+^ Tregs (indicated by white arrows) in tumour tissues. (**B**) Kaplan–Meier survival curves of CRC patients in the Renji cohort. The samples were grouped based on the number of Tregs and TNFR2^+^CCR8^+^ Tregs. The median cutoff for TNFR2^+^CCR8^+^ Tregs was 11 cells/mm^2^ in CRC patients. HR, hazard ratio. (**C**) Kaplan–Meier survival curves of GC patients in the Renji cohort. The samples were grouped based on the number of Tregs and TNFR2^+^CCR8^+^ Tregs. The median cutoff for TNFR2^+^CCR8^+^ Tregs was 8 cells/mm^2^ in GC patients. (**D**) The working model of this study. TNF-α, which is highly expressed in the TME, not only upregulates the immunosuppressive function of Tregs but also activates the NF-κB signalling pathway in Tregs through TNFR2. In this process, P65 (an NF-κB subunit) and FOXP3 bind to the promoter region of CCR8, thus enhancing the transcription of CCR8. Furthermore, TNFR2^+^CCR8^+^ Tregs are recruited into the TME by CCL18^+^ macrophages through the CCL18/CCR8 chemotaxis axis. In addition, the aggregation of a large number of TNFR2^+^CCR8^+^ Tregs in the TME destroys the therapeutic effect of anti-PD1 therapy. TAM, tumour-associated macrophage.

Based on the immunofluorescence staining results, 54 CRC patients and 60 gastric cancer (GC) patients were divided into the following two groups: a high TNFR2^+^CCR8^+^ Treg infiltration group and a low TNFR2^+^CCR8^+^ Treg infiltration group (Figure 8A). In CRC patients, high levels of tumour-infiltrating TNFR2^+^CCR8^+^ Tregs, but not total Tregs, were associated with an adverse prognosis (Figure 8B), indicating that TNFR2^+^CCR8^+^ Tregs might play important roles in the progression of CRC. The same conclusion could be drawn for GC patients (Figure 8C). These results suggested that tumour-infiltrating TNFR2^+^CCR8^+^ Tregs might have important impacts on the progression and treatment of gastrointestinal tumours.

## Discussion

Our study revealed the relationship between two markers (TNFR2 and CCR8) of tumour-infiltrating Tregs and suggested TNFR2^+^CCR8^+^ Tregs as a potential immunotherapeutic target to enhance antitumour immunity and strengthen anti-PD1 treatment efficacy in CRC patients. Using human tumour samples and murine subcutaneous tumour models, we demonstrated that TNF-α-induced TNFR2^+^CCR8^+^ Tregs were more immunosuppressive and able to dampen antitumour T cell responses, thus promoting tumour growth.

Many studies have illustrated that a high level of tumour-infiltrating Tregs in the TME leads to immune escape (Kondĕlková et al., 2010; Xie et al., 2019). The precise phenotype, function, and regulatory mechanism of activated suppressive Tregs, which have a major impact on tumour immunotherapy, are urgently needed. CCR8^+^ Tregs, the major activated suppressive Treg subgroup reported in our previous work, play a critical role in the TME. CCL1 upregulated the expression of CCR8 in Tregs via the STAT3 signalling pathway and promoted the inhibitory function of Tregs (Barsheshet et al., 2017). However, the expression of CCL1 is extremely low in a variety of tumour tissues (Korbecki et al., 2020). Therefore, CCR8 expression might not be regulated by CCL1 in the TME, and the actual regulatory mechanism of CCR8 expression remains unknown. Here, we provided the first in-depth analysis showing that in CRC, TNF-α/TNFR2 upregulated the expression of CCR8 in Tregs in a P65- and FOXP3-dependent manner.

It has been reported that TNF-α not only increases FOXP3 expression in Tregs but also enhances the suppressive capacity of Tregs in both mice and humans (Yang et al., 2021). Other studies have found that TNF-α/TNFR2 is crucial for sustaining FOXP3 expression and maintaining the stability of murine Tregs in an inflammatory environment (Chen et al., 2013). In this study, we revealed that TNF-α not only strengthened the immunosuppressive functions of Tregs but also increased the expression of CCR8, which played a vital role in the chemotaxis of Tregs. A previous study showed that two CCL18 receptors, PITPNM3 and CCR8, were highly expressed in tumour-infiltrating Tregs. Tregs enter the TME mainly through the chemotaxis of PITPNM3–CCR8 (Su et al., 2017). However, how CCR8 functions as a chemokine receptor in Tregs remains unclear. We observed that CCL18 was highly expressed in tumour-infiltrating CD68^+^ macrophages, which attracted Tregs via the CCL18/CCR8 interaction, so we speculated that CCL18^+^ macrophages could lead to the accumulation of CCR8^+^ Tregs in the TME. However, more specific *in vivo* experiments are needed, such as the construction of CCL18 or CCR8 knockout mice. The accumulated CCR8^+^ Tregs gradually formed a highly immunosuppressive microenvironment within the tumour tissues. Anti-TNFR2 mAb treatment might destroy the chemotactic attraction between CCL18^+^ macrophages and CCR8^+^ Tregs, leading to a reduction in tumour-infiltrating CCR8^+^ Tregs.

Recently, several studies have reported that therapies targeting CCR8^+^ Tregs show high efficacy in the treatment of colon cancer, non-small cell lung cancer, and melanoma; these findings highlight the efficacy and safety of targeting CCR8 for the depletion of tumour-promoting Tregs in combination with anti-PD1 therapy (Campbell et al., 2021; Van et al., 2021; Kidani et al., 2022). However, the specific role of CCR8 in Tregs is still controversial (Whiteside et al., 2021; Wang et al., 2022), and the specific mechanism by which CCR8^+^ Tregs are regulated remains unclear. In this work, we mainly studied the upstream signals for CCR8^+^ Treg induction, revealed the transformation and aggregation processes of CCR8^+^ Tregs in the TME, and eventually proposed TNFR2 as a potential target for this subgroup.

ICIs (anti-PD1 or anti-PDL1 mAbs) have provided substantial benefits for patients with several cancers. However, the overall response rate to anti-PD1 or anti-PDL1 mAb therapy rarely exceeds 40% (Yarchoan et al., 2017). A large number of tumour-infiltrating Tregs are considered to contribute to resistance to anti-PD1 therapy and are associated with worse overall survival (Jenkins et al., 2018; Upadhaya et al., 2021). Consistently, we found that anti-PD1 mAb treatment could induce TNFR2^+^CCR8^+^ Treg infiltration, hindering the therapeutic effect of anti-PD1 therapy. Under anti-PD1 mAb stimulation, CD8^+^ T cells released more TNF-α, which contributed to the accumulation and immunosuppressive functions of CCR8^+^ Tregs. Our results also demonstrated that the combination of anti-TNFR2 mAb and anti-PD1 mAb had better therapeutic efficacy than either antibody alone in both anti-PD1-responsive and anti-PD1-unresponsive CRC mouse models.

In summary, our findings provide insights into the regulatory mechanisms of CCR8^+^ Tregs, and we propose TNFR2 as a promising therapeutic target for the treatment of CRC.

## Materials and methods

### Patient samples

After approval from the hospital ethics committee, fresh patient samples were collected from the Department of Gastrointestinal Surgery, Renji Hospital, School of Medicine, Shanghai Jiao Tong University. The clinical criteria for patient recruitment were as follows: (i) the patients had no autoimmune disorders or other primary malignant tumours; (ii) the patients had not been treated with chemotherapy, radiation, or any other antitumour medicine before tumour resection; and (iii) the patients had complete clinical information, postoperative pathological diagnoses, and follow-up data. Finally, 34 CRC tissues with paired adjacent tissues were collected for flow cytometry analysis. A total of 54 CRC and 60 GC samples were analysed with immunofluorescence staining. All patients agreed with the experimental design and provided written informed consent. The study protocols were approved by the ethical review community of the Shanghai Jiao Tong University School of Medicine, Renji Hospital (no. 2017-114-CR-02).

### Preparation of single-cell suspensions

Tissues were collected from primary CRC patients who underwent surgery. All tissue specimens were cut into smaller pieces and incubated in Dulbecco's Modified Eagle's Medium (Cat# 11054001, Gibco) containing 10% fetal bovine serum (FBS) (200 U/ml), type IV collagenase (Cat# C5138, Sigma), and 0.1 mg/ml DNase I (Cat# D5025, Sigma) for 30 min at 37°C on a shaker (200 rpm). The dissociated cell suspensions were then passed through 70-μm cell strainers and 40-μm cell strainers (Cat# 352340 and Cat# 352360, Falcon) to obtain single-cell suspensions. The single-cell suspensions were washed twice with PBS. Trypan blue was used to detect cell viability. When the cell viability was ˃90%, the cells were used in subsequent experiments. PBMCs were obtained from patients before surgery. PBMCs were isolated by density gradient centrifugation (speed at 2000 rpm; acceleration ramp 3 and braking ramp 0; at room temperature) for 20 min using Ficoll-Paque (Cat# 17-1440-02, GE Healthcare).

### Flow cytometry

Fresh tumour tissues were collected during surgery. Single-cell suspensions were prepared as described above. The cells were collected and transferred to 96-well plates or flow tubes. Single cells were stained with different antibodies (antibody information is shown in Supplementary Table S4) in PBS containing 2% FBS for 30 min at 4°C in the dark. The cells were then fixed and permeabilized with fixation/permeabilization concentrate (Cat# 00-5123-43, eBioscience) for 40 min at room temperature in the dark. Intracellular targets were stained with the corresponding antibodies for 40 min at room temperature, followed by two washes with permeabilization buffer (Cat# 00-8333-56, eBioscience). For determination of cytokine expression, cells were stimulated with 0.25 μg/ml PMA (Cat# P-8139, Sigma), 0.25 μg/ml ionomycin (Cat# I-0634, Sigma), and 1:1000 Golgistop (Cat# 554724, BD Biosciences) for 4 h at 37°C. A BD LSRFortessa X-20 cell analyser and a BD FACSAria III (BD Biosciences) were used for flow cytometry and FACS, respectively. Data analysis was performed with FlowJo v10 (FlowJo LLC).

### *In vitro* cell proliferation assay

Human CRC tissues were processed into single-cell suspensions, and tumour-infiltrating lymphocytes were isolated from the suspensions as described above. The TNFR2^+^CCR8^+^ and TNFR2^−^CCR8^−^ Tregs were sorted from the tumour-infiltrating lymphocytes by a BD FACSAria III (BD Biosciences). Human peripheral CD8^+^ T cells were sorted from blood samples of healthy volunteers. The CD8^+^ T cells were labelled with CellTrace Violet following the protocol of the CellTrace Violet Cell Proliferation Kit (Cat# C34557, Invitrogen). CTV-labelled CD8^+^ T cells were either cultured alone or cocultured with TNFR2^+^CCR8^+^or TNFR2^−^CCR8^−^ Tregs at different ratios. The cell mixtures were stimulated with anti-CD3/CD28 DynaBeads for three days. Cell proliferation was measured by flow cytometry.

### CUT&RUN assay

The CUT&RUN Assay Kit (Cat# 86652, Cell Signaling Technology) was used as instructed by the manufacturer. Tregs were sorted from blood samples of healthy donors. Tregs were stimulated with TNF-α (15 ng/ml) for 2 h. Then, the cells were pelleted at 350× *g* for 5 min at 4°C. The supernatant was discarded, and the cells were washed with 1 ml of cold PBS, bound to concanavalin A-coated magnetic beads, permeabilized with wash buffer (20 mM HEPES, pH 7.5, 150 mM NaCl, 0.5 mM spermidine, and protease inhibitor) containing 0.05% digitonin (Dig Wash), and incubated with specific primary antibodies, including anti-FOXP3 (Cat# PA1-806, Invitrogen), anti-P65 (Cat# D14E12, Abcam), and rabbit IgG (Cat# 66362, Cell Signaling Technology) antibodies, overnight at 4°C. The cell-bead slurry was washed twice with Dig Wash, incubated with Protein A-MNase (pA-MN) for 1 h at 4°C, and then washed twice with Dig Wash. The slurry was placed on an ice-cold block and incubated with Dig Wash containing 2 mM CaCl_2_ to activate pA-MN digestion. After 5 min of digestion, the reactions were stopped by the addition of 150 μl of 2× Stop Buffer. The samples were centrifuged for 5 min at 16000× *g*, and the supernatants were transferred to new microfuge tubes. DNA fragments were purified via a DNA Purification Buffers and Spin Columns Kit (Cat# 14209, CUT&RUN, Cell Signaling Technology).

Quality assessment was performed using FastQC (v.0.11.9) and MultiQC (v1.12). Fastq files were then trimmed using Trim Galore and aligned to the GRCh38 genome using Bowtie 2. Then, SAMtools (v.1.1) was used to sort the bam file. Peak calling was performed with MACS2 (v.2.1.3.3), and data visualization was performed using IGV with the R language.

### MC38/CT26 subcutaneous tumour model

Mouse MC38/CT26 CRC cells were digested with trypsin and processed into single cells, as described previously. The single-cell solutions were centrifuged, and the supernatant was removed thoroughly. The pellets were resuspended in 1× PBS and centrifuged for 10 min at 400× *g* at room temperature. For the tumour growth assay, male *Tnfr2*^−/−^ and WT mice aged 6–8 weeks were used as recipients. For each mouse, 100 μl of MC38 cells (5 × 10^5^ cells) was subcutaneously injected. For the drug administration experiment, 6- to 8-week-old WT mice were used as recipients. MC38 cells were diluted to 2 × 10^6^ cells/ml, and CT26 cells were diluted to 5 × 10^6^ cells/ml. One hundred microlitres of MC38 cells (2 × 10^5^ cells per mouse) or CT26 cells (5 × 10^5^ cells per mouse) were subcutaneously injected. Three days after the subcutaneous injection of MC38 or CT26 cells, the volume of the transplanted tumour was measured every two days. Once the volume of the tumours reached 100 mm^3^, the mice were treated with 10 mg/kg isotype control, anti-TNFR2 mAb, anti-PD1 mAb, or anti-TNFR2 mAb combined with anti-PD1 mAb every two days. Flow cytometry was used to detect the differences among immune cell subsets across tissues.

### Statistical analysis

The *t*-test, ANOVA, Mann–Whitney U test, and Chi-square test were used for statistical analysis when appropriate. Correlation scores were obtained using the Spearman test. Statistical analyses were performed using SPSS 23.0 and GraphPad Prism 8.0. Data are presented as mean ± standard deviation (SD) unless otherwise stated. Differences with a *P*-value of <0.05 were considered significant.

## Supplementary Material

mjad067_Supplemental_File

## Data Availability

The data that support the findings of this study are available from the corresponding author upon reasonable request.

## Notes

**Author contributions:** Y.G.: conceptualization, performing experiments, writing the original draft, reviewing, and editing. F.X.: project administration, performing experiments, reviewing, and editing. X.L.: project administration and raw data collection and analysis. Y.Z.: developing specific software. S.K.: performing experiments and writing the original draft. N.L.: validation, supervision, and raw data collection and analysis. Z.W.: validation. G.Y.: data curation. Y.S.: raw data collection, analysis, and curation. J.C.: developing software and writing the original draft. D.L.: supervision and validation. C.Z.: supervision. Z.Z.: project administration. H.L.: supervision. G.Z.: funding acquisition and supervision. B.L.: funding acquisition and supervision. W.Z.: supervision, reviewing, and editing.
