# Supplementary material for: Blockade of TNF-α/TNFR2 signalling suppresses colorectal cancer and enhances the efficacy of anti-PD1 immunotherapy by decreasing CCR8+T regulatory cells
Source: J Mol Cell Biol. 2023 Nov 3;16(6):mjad067. doi: 10.1093/jmcb/mjad067 (PMC11587560; doi:10.1093/jmcb/mjad067)
Supplement: mjad067_Supplemental_File [file mjad067_supplemental_file.docx]

**Supplementary Methods**

**Cell sorting and cultures**

Whole umbilical cord blood (UCB) was kindly donated by Renji Hospital, and adult peripheral blood was obtained from healthy donors. CD4^+^CD25^low^CD127^high^CD45RA^+^ naïve T cells were isolated from mononuclear cells enriched from UCB or adult blood. Tregs were differentiated into induced Tregs (iTregs) in X-VIVO (Cat# 04-418Q, Lonza, Malkersville, USA) medium with 10% foetal bovine serum (FBS, Cat# 10100147, Invitrogen, Maltham, USA), 1% GlutaMAX (Cat# 35050061, Gibco, New York, USA), 1% sodium pyruvate (Cat# 11360070, Gibco), 1% minimum essential medium with nonessential amino acids (Cat# 11140050, Gibco), 1% penicillin‒streptomycin (Cat# 15140122, Gibco), 100 U/ml rhIL-2 (Cat# 202-IL, R&D Systems, Minneapolis, USA), and 5 ng/ml transforming growth factor-β (rhTGF-β, 5 ng/ml, Cat# 7754-BH, R&D ­Systems) in the presence of Dynabeads Human T-activator CD3/CD28 (Cat# MBS-C001, Gibco) at a bead-to-cell ratio of 1:4. Approximately 7 days later, the differentiation efficiency reached at least 90% and could be used for analysis.

**Treg stimulation**

Human peripheral Tregs, defined as CD4^+^CD25^+^CD127^−^ T cells, were sorted by FACSAria II (BD Biosciences) from the blood samples of healthy donors. Tregs were stimulated with TTCS, NTCS (autologous of TTCS), TTCS with a neutralizing antibody against human TNF-α (5, 10 μg/ml) (Cat# AF-210-SP, R&D Systems), or NTCS with rhTNF-α (5, 15 ng/ml) (Cat# [10291-TA](https://www.rndsystems.com/cn/products/recombinant-human-tnf-alpha-hek293-expressed-protein-cf_10291-ta), R&D Systems) for 48 h in a humidified incubator at 37°C. After stimulation, the cells were collected for flow cytometry and western blotting analyses. For the signalling pathway inhibition experiments, Tregs were pretreated with anti-TNFR1 mAb (5 μg/ml), anti-TNFR2 mAb (5 μg/ml), ruxolitinib (3.3 nM) to block the STAT3 signalling pathway, PDTC (20 μM) to block the NF-κB signalling pathway, and Bis-I (27 nM) to block the PKC signalling pathway. Tregs were then stimulated with TTCS or TNF-α (15 ng/ml) for 48 h, followed by flow cytometry and western blotting analyses.

**Tissue culture and preparation of tumor tissue culture supernatant (TTCS) and normal tissue culture supernatant (NTCS)**

All tissue specimens were cut into small pieces and cultured in 1 ml Lonza X-VIVO medium supplemented with 10% FBS, 1% GlutaMAX, 1% sodium pyruvate, 1% minimum essential medium with nonessential amino acids, 1% penicillin-streptomycin in a humidified incubator at 37°C for 48 hrs. The supernatants were collected by centrifugation.

**Lentivirus infection of human primary Treg cells**

Short hairpin RNA (shRNA) was constructed following the protocol provided by the manufacturer of the pLKO.1 vector. Human CD4^+^CD25^low^CD127^high^CD45RA^+^ naive T cells, isolated from healthy donors’ PBMCs, were differentiated into iTregs using anti-CD3/CD28 DynaBeads in the presence of rhIL-2 and rhTGF-β. The iTregs were cultured in X-VIVO medium supplied with 10% FBS for 7 days. In 24 well plates, 1 μg/ml anti-CD3/CD28 DynaBeads were added to each well and incubated at 37°C for 72 hrs before lentivirus infection. To activate and maintain the proliferative status of the human iTregs, iTreg cells were seeded, 0.1×10^6^ cells per well, into the CD3/CD28 DynaBeads pre-coated plates and incubated for 48 hrs. When the concentration of activated iTreg cells reached 1 million cells/ml, the lentivirus at the multiplicity of infection, 100 U/ml rhIL-2 (maintaining Treg cells), and 8 ng/ml polybrene (Cat# H9268, Sigma) (promoting virus infection) were added into each well. The cell culture plates were centrifuged at 2000 rpm for 2 mins to promote contact between the virus and cells. After 20-24 hrs infection, the plates were centrifuged and the media were changed to normal culture media. Four days after infection, the infection efficiency was determined by flow cytometry or flow sorter. The GFP-positive living cells were selected for subsequent experiments.

**Luciferase assay**

The pGL3 luciferase reporter vectors, both control and expression vectors, for NF-κB were purchased from Addgene (Watertown, USA). A 2000 bps human CCR8 promoter was inserted into pGL3 vectors. HEK293T cells were transiently transfected, in Lipofectamine 2000 solution (Cat# 11668027, Invitrogen), with CCR8-pGL3 (0.25 μg per well), pGL3 (0.05 μg per well) and expression vectors (0.5 μg per well), respectively. Promoter activity was measured with the firefly-Luciferase Reporter Assay System (Cat# RG027, Beyotime, Shanghai, China) according to the manufacturer’s instructions.

**Luminex assay**

Sample solutions (tumor tissue culture supernatant and normal tissue culture supernatant) were collected and placed into 96 well plates. Highly specific capture antibodies coupled with different fluorescent-labeled magnetic beads were used in this experiment. The magnetic beads were mixed with the sample solutions in the 96 well plates and incubated for 2 hrs at RT. The target proteins would bind to the capture antibodies on the beads. After washing, the beads were incubated with biotinylated target-specific detector antibodies for 1 hr at RT. Next, excess biotinylated detector antibodies were removed. The streptavidin-conjugated fluorescent protein, R-Phycoerythrin (SAV-RPE), was added to the beads and incubated for 30 mins at RT. SAV-RPE would bind to the biotinylated detector antibodies. After removing free SAV-RPE, the beads were analyzed with a Luminex detection system (ThermoFisher). The quantities of antigens were measured by the fluorescence intensity.

**Transwell** **migration assay**

T cell migration was evaluated using 24-well Transwell plates (Cat# 3402, 6.5 mm, 5.0 μm, Corning, New York, USA). Fresh CCR8^+^ Tregs and CCR8^−^ Tregs were isolated via flow cytometric sorting. CCR8^+^ Tregs and CCR8^−^ Tregs were maintained in X^-^VIVO culture medium containing 10% FBS. The Tregs stock was diluted to 5×10^5^ cells/ml and a small portion (100 μl) of this suspension was placed into the top chamber of the 24-well Transwell plate. CCL18 (100 ng/ml, Cat# 394-PA-050/CF, R&D System) and 500 μl medium were added into the bottom chamber. After incubation with 5% CO_2_ at 37°C for 90 mins, the apical cavity was removed and the number of Treg cells migrated to the bottom cavity was counted under the microscope.

**Real-time PCR**

Total RNAs extractions were performed using TRIzol (Cat# 15596018, ThermoFisher, Waltham, USA) following the manufacturer’s instructions. The concentrations of total RNAs were measured with NanoDrop One (ThermoFisher). Total RNAs were reverse transcribed to cDNAs using Hiscript QRT supermix (+ gDNA WIPER) (Cat# R123-01, Vazyme, Nanjing, China). The cDNAs were diluted with ddH2O at the ratio of 1:5, which was adjusted according to the concentration of extracted RNA. The real-time quantitative PCRs were carried out using the SYBR premix extapaquKit (Cat# RR420A, Takara, Japan). With β-actin as an internal reference, each sample was tested in triplicates. The formula for calculating the relative expression of a gene was RQ = 2^-△△Ct^.

**Western Blot**

Cells and tissues were lysed with RIPA buffer (Cat# 89901, ThermoFisher). Protein concentrations were measured with NanoDrop On. Western blot assays were performed with 8% SDS-PAGE gels, onto which an equivalent amount of cell lysate proteins was loaded. Proteins were transferred to nitrocellulose (NC) membranes (Cat# HATF00010, Millipore, USA) and the membranes were blocked with 5% skimmed milk. Human FOXP3, P65, and p-P65 proteins were detected with anti-FOXP3, anti-P65, and anti-p-P65 antibodies (antibody information was shown in supplementary table 4), respectively. This was followed by the incubation of HRP-conjugated secondary antibodies. The protein bands were visualized with immobilization Western HRP chemiluminescence (Cat# WBKLS0100, Millipore).

**Immunofluorescence**

Patient tumor samples were fixed with formalin and paraffin. The formalin-fixed paraffin-embedded (FFPE) tissue blocks were cut into thin slices and punched onto glass slides. Pathological diagnosis of these slices was confirmed by two senior pathologists through the review of H&E-stained slides. Tissue slides were subsequently placed into xylene, 100% ethanol, 80% ethanol, 75% ethanol, and distilled water. Tissue sections were placed in EDTA antigen repair buffer (ph8.0) (Cat# E1170, Solarbio, Beijing, China) at 4°C for antigen retrieval. Slides were incubated in 3% hydrogen peroxide solution, at RT for 25 mins in dark, to block endogenous peroxidase activity. The 3% bovine serum albumin (Cat# PH0501, scientific phygene, Fuzhou, China) was added to block non-specific antigens. The slides were incubated with corresponding primary antibodies in a wet box at 4° C overnight. Slides were then incubated with HHRP-labeled secondary antibodies at RT for 50 mins. Slides were incubated with If555 tyramide (Cat# G1233, Servicebio, Wuhan, China), followed by heating in a microwave. The nuclei were stained with DAPI. Slides were sealed with an anti-fluorescence quenching sealing agent. Stained sections were visualized with microscopy. In a two-category Immunocore analysis, patients were dichotomized into the high- and low-density group according to the median number of stained cells. As a result, the median cutoff for TNFR2^+^CCR8^+^ Treg was 8 /mm^2^ in GC and 11 /mm^2^ in CRC.

**ELISA**

Human CRC tissues and mouse tumor tissues were collected. Tumor tissues were lysed and the supernatants were collected for ELISA analysis. Concentrations of TNF-α in the supernatants were determined using ELISA kits (Cat# JL10208, R&D Systems) according to the manufacturer’s instructions.

**Cell line-derived xenograft model**

CD14^+^ cells were isolated from PBMCs and purified by positive selection with human CD14 magnetic particles (Cat# 130-110-517, Miltenyi, Bergisch Gladbach, Germany) to generate dendritic cells (DCs), as described previously (Brandes et al., 2005). DCs were differentiated from CD14^+^ monocytes in the presence of 50 ng/μl granulocyte-macrophage colony-stimulating factor (GM-CSF, Cat# P04141, Peprotech, Inc. Rocky Hill, USA) and 20 ng/μl IL-4 (Ca# 204-IL, R&D Systems) for 5 days.

DCs from healthy donors were incubated with irradiated apoptotic HCT116 cells at a ratio of 1:5 (DC vs. HCT116) for 24 h. Allogenic magnetic bead (Cat# 130-045-201, Miltenyi, Germany)-purified peripheral CD8^+^ T cells (2×10^5^ cells/well in 96-well plates) were activated by incubation with tumour‐loaded DCs (2×10^4^ cells/well) in X-VIVO medium containing rh IL-2 (20 IU/ml), rh IL-7 (10 ng/ml, Cat# 10531-IR-050, R&D Systems), and anti-CD3/CD28 DynaBeads (2 µg/ml) antibodies for one week. Consequently, we harvested tumour-specific CD8^+^ T cells as described in a previous report (Shan et al., 2022).

A total of 1×10^6^ CRC cells (HCT116) in 100 μl of PBS were subcutaneously injected into the axillary tissues of male NOD/SCID mice (6–8 weeks). Tumour‐specific CD8^+^ T cells from healthy donors and TNF-α-induced TNFR2^+^CCR8^+^ Tregs generated from allogenic blood were sorted by FACS as described above. Then, 1×10^6^ polyclonal-stimulated (anti-CD3/CD28 DynaBeads) tumour‐specific CD8^+^ T cells were cocultured with or without 1×10^6^ TNFR2^+^CCR8^+^ Treg or TNFR2^−^CCR8^−^ Treg cells at a ratio of 1:2 for 24 h. The cell mixtures, in 100 μl of PBS, were subsequently injected into the tails of mice on day 5 after tumour cell inoculation. Tumour size was measured every 2 days by two independent observers using callipers. Tumour volume was calculated based on three perpendicular measurements. Once the mice were sacraficed, tumours were photographed before paraformaldehyde fixation for immunohistochemical staining.

**Supplementary table S1**

**Characteristics of CRC patients for FACS analysis**

|  | colorectal cancer | | |  |
| --- | --- | --- | --- | --- |
|  | number | | percentage | |
| Total | 34 | |  | |
| Gender |  | |  | |
| male | 13 | | 38.24% | |
| female | 21 | | 61.76% | |
| Age |  | |  | |
| <65 | 12 | | 35.29% | |
| >65 | 22 | | 64.71% | |
| Tumor stage |  | |  | |
| T1 | 8 | | 23.50% | |
| T2 | 9 | | 26.47% | |
| T3 | 7 | | 20.59% | |
| T4 | 10 | | 29.41% | |
| Lymph node metastasis |  | |  | |
| N0 | 14 | | 41.18% | |
| N1 | 20 | | 58.82% | |
| Distant metastasis |  | |  | |
| M0 | 25 | | 73.53% | |
| M1 | 9 | | 26.47% | |
| AJCC stage |  | |  | |
| I | 8 | | 23.53% | |
| II | 8 | | 23.53% | |
| III | 9 | | 26.47% | |
| IV | 9 | | 26.47% | |
| Histological Grading | |  |  | |
| Well differentiated | | 7 |  | |
| Moderately differentiated | | 15 |  | |
| poorly differentiated | | 12 |  | |

**Supplementary table S2**

**Characteristics of CRC patients for IF analysis**

|  | colorectal cancer | | |
| --- | --- | --- | --- |
|  | number | | percentage |
| Total | 54 | | 100% |
| Gender |  | |  |
| male | 27 | | 50.00% |
| female | 27 | | 50.00% |
| Age |  | |  |
| <65 | 25 | | 46.30% |
| >65 | 29 | | 53.70% |
| Tumor stage |  | |  |
| T1 | 7 | | 12.96% |
| T2 | 14 | | 25.93% |
| T3 | 11 | | 20.37% |
| T4 | 22 | | 40.74% |
| Lymph node metastasis |  | |  |
| N0 | 20 | | 37.04% |
| N1 | 34 | | 62.96% |
| Distant metastasis |  | |  |
| M0 | 46 | | 85.19% |
| M1 | 8 | | 14.81% |
| AJCC stage |  | |  |
| I | 16 | | 29.63% |
| II | 13 | | 24.07% |
| III | 17 | | 29.63% |
| IV | 8 | | 16.67% |
| Histological Grading |  | |  |
| Well differentiated | | 17 | 31.38% |
| Moderately differentiated | | 20 | 37.03% |
| poorly differentiated | | 17 | 31.48& |
| TNFR2+CCR8+Treg | |  |  |
| High | | 27 | 50.00% |
| Low | | 27 | 50.00% |

**Supplementary table S3**

**Characteristics of GC patients for IF analysis**

|  | gastric cancer | | |
| --- | --- | --- | --- |
|  | number | | percentage |
| Total | 60 | | 100% |
| Gender |  | |  |
| male | 37 | | 61.67% |
| female | 23 | | 38.33% |
| Age |  | |  |
| <65 | 35 | | 58.33% |
| >65 | 25 | | 41.67% |
| Tumor stage |  | |  |
| T1 | 10 | | 16.67% |
| T2 | 16 | | 26.67% |
| T3 | 13 | | 21.67% |
| T4 | 21 | | 35.00% |
| Lymph node metastasis |  | |  |
| N0 | 24 | | 40.00% |
| N1 | 36 | | 60.00% |
| Distant metastasis |  | |  |
| M0 | 50 | | 83.33% |
| M1 | 10 | | 16.67% |
| AJCC stage |  | |  |
| I | 14 | | 23.33% |
| II | 14 | | 23.33% |
| III | 22 | | 36.67% |
| IV | 10 | | 16.67% |
| Histological Grading | |  |  |
| Well differentiated | | 20 | 33.33% |
| Moderately differentiated | | 25 | 41.67% |
| poorly differentiated | | 15 | 25.00% |
| TNFR2+CCR8+Treg | |  |  |
| High | | 30 | 50% |
| Low | | 30 | 50% |

**Supplementary table S4**

**Antibody list**

| antibody | supplier,clone | application |
| --- | --- | --- |
| P65 | CST, D14E12 | WB |
| pP65 | CST, 93H1 | WB |
| Beta-actin | Proteintech, 2D4H5 | WB |
| CD8 | CST, 53-6.7 | IF |
| FOXP3 | Biolegend, 433H | IF |
| TNFR2 | Affinity | IF |
| CCR8 | Affinity | IF |
| CD3 | Biolegend,17A2 | FACS |
| CD25 | Biolegend,M-A251 | FACS |
| CD8 | Biolegend,SK1 | FACS |
| FOXP3 | Biolegend,259D | FACS |
| TNFR2 | R&D, TR7554 | FACS |
| CCR8 | BD, 433H | FACS |
| CTLA4 | Biolegend, L3D10 | FACS |
| IFN-γ | Biolegend, GIR-208 | FACS |
| TNF-α | Biolegend, MAb11 | FACS |
| CD68 | Biolegend, Y1/82A | FACS |
| CCL18 | Miltenyi, REA487 | FACS |
| CD45 | Biolegend,30-F11 | FACS |
| CD4 | Biolegend, RM4-5 | FACS |
| CD8 | Biolegend,Ly-3 | FACS |
| FOXP3 | Biolegend, MF-14 | FACS |
| TNFR2 | eBioscience, TR75-89 | FACS |
| IFN-γ | Biolegend, XMG1.2 | FACS |
| TNF-α | R&D, DY210 | ELISA |
| FOXP3 | Biolegend, 433H | ChIP |
| P65 | CST, D14E12 | ChIP |
| rhTNF-α | R&D, 210-TA | Bioactivity |
| rmTNF-α | R&D, 410-MT | Bioactivity |
| CCL18 | R&D, 394-PA | Bioactivity |
| anti-TNF-α mAb | R&D | block |
| anti-TNFR1 mAb | R&D | block |
| anti-TNFR2 mAb | R&D | block |
| IgG1 | R&D | block |
| anti-PD1 mAb | BioXcell, RMP1-14 | block |
| anti-TNFR2 mAb | Biolegend, TR75-54.7 | block |
| IgG2a | BioXcell, 2A3 | block |
| ruxolitinib | selleck | inhibitor |
| bisindolylmaleimide I | selleck | inhibitor |
| PDTC | selleck | inhibitor |

**Supplementary table S5**

**Primers List**

| application | gene |  | sequence |
| --- | --- | --- | --- |
| quatification | Human genes  β-ACTIN | F | CATGTACGTTGCTATCCAGGC |
|  |  | R | CTCCTTAATGTCACGCACGAT |
|  | CCR4 | F | CCCACGGATATAGCAGACACC |
|  |  | R | GTGCAAGGCTTGGGGATACT |
|  | CCR5 | F | TTCTGGGCTCCCTACAACATT |
|  |  | R | TTGGTCCAACCTGTTAGAGCTA |
|  | CCR6 | F | TTCAGCGATGTTTTCGACTCC |
|  |  | R | GCAATCGGTACAAATAGCCTGG |
|  | CCR7 | F | TGAGGTCACGGACGATTACAT |
|  |  | R | GTAGGCCCACGAAACAAATGAT |
|  | CCR8 | F | GTGTGACAACAGTGACCGACT |
|  |  | R | CTTCTTGCAGACCACAAGGAC |
|  | CCR9 | F | ATGTCAGGCAGTTTGCGAG |
|  |  | R | TGCAGTACCAGTAGACAAGGAT |
|  | CXCR3 | F | CCACCTAGCTGTAGCAGACAC |
|  |  | R | AGGGCTCCTGCGTAGAAGTT |
|  | CXCR4 | F | ACTACACCGAGGAAATGGGCT |
|  |  | R | CCCACAATGCCAGTTAAGAAGA |
|  | CXCR5 | F | CACGTTGCACCTTCTCCCAA |
|  |  | R | GGAATCCCGCCACATGGTAG |
|  | TNF-α | F | CCTCTCTCTAATCAGCCCTCTG |
|  |  | R | GAGGACCTGGGAGTAGATGAG |
|  | Mouse genes |  | ACGTCACGATGACCGACTACT |
|  | Ccr8 | F | ACGAGGACTAAGATGACCAGG |
|  |  | R |  |
|  | Foxp3 | F | ACGTCACGATGACCGACTACT |
|  |  | R | ACGAGGACTAAGATGACCAGG |
|  | Cd25 | F | CAAGAACGGCACCATCCTAAA |
|  |  | R | TCCTAAGCAACGCATATAGACCA |
|  | Tnfr2 | F | ACACCCTACAAACCGGAACC |
|  |  | R | AGCCTTCCTGTCATAGTATTCCT |
|  | Ctla4 | F | CATGGTGTCGCCAGCTTTC |
|  |  | R | GGTAATCTAGGAAGCCCACTGTA |
| ChIP | CCR8   (482-487) | F | CATCGGCTCACCATTGATCTTTGCTT |
|  |  | R | CATAAAGGAACTGGGTTTTCCCCAA |
|  | CCR8  (542-552) | F | ATACCTGAGGGCCCTGAGTT |
|  |  | R | TCCCCTCACCATCATTCATGT |
| shRNA | FOXP3 | F | CCGG AGCTGGAGTTCCGCAAGAAACCTCG AG GTTTCTTGCGGAACTCCAGCTTTTTTG |
|  |  | R | AATTCAAAAA AGCTGGAGTTCCGCAAGAA AC CTCGAG GTTTCTTGCGGAACTCCAGCT |
| Genotype identification | KO | F | TATTCTCCTGGCAGTTGGAGTCTG |
|  |  | R | CTAGGGATATAAGCAGAACGTGGC |
|  | WT | F | AGGCTGGACTTGATCTCAGCATC |
|  |  | R | AGCTCACAGAATCCGAGAGACTC |

F: forward, R: reverse


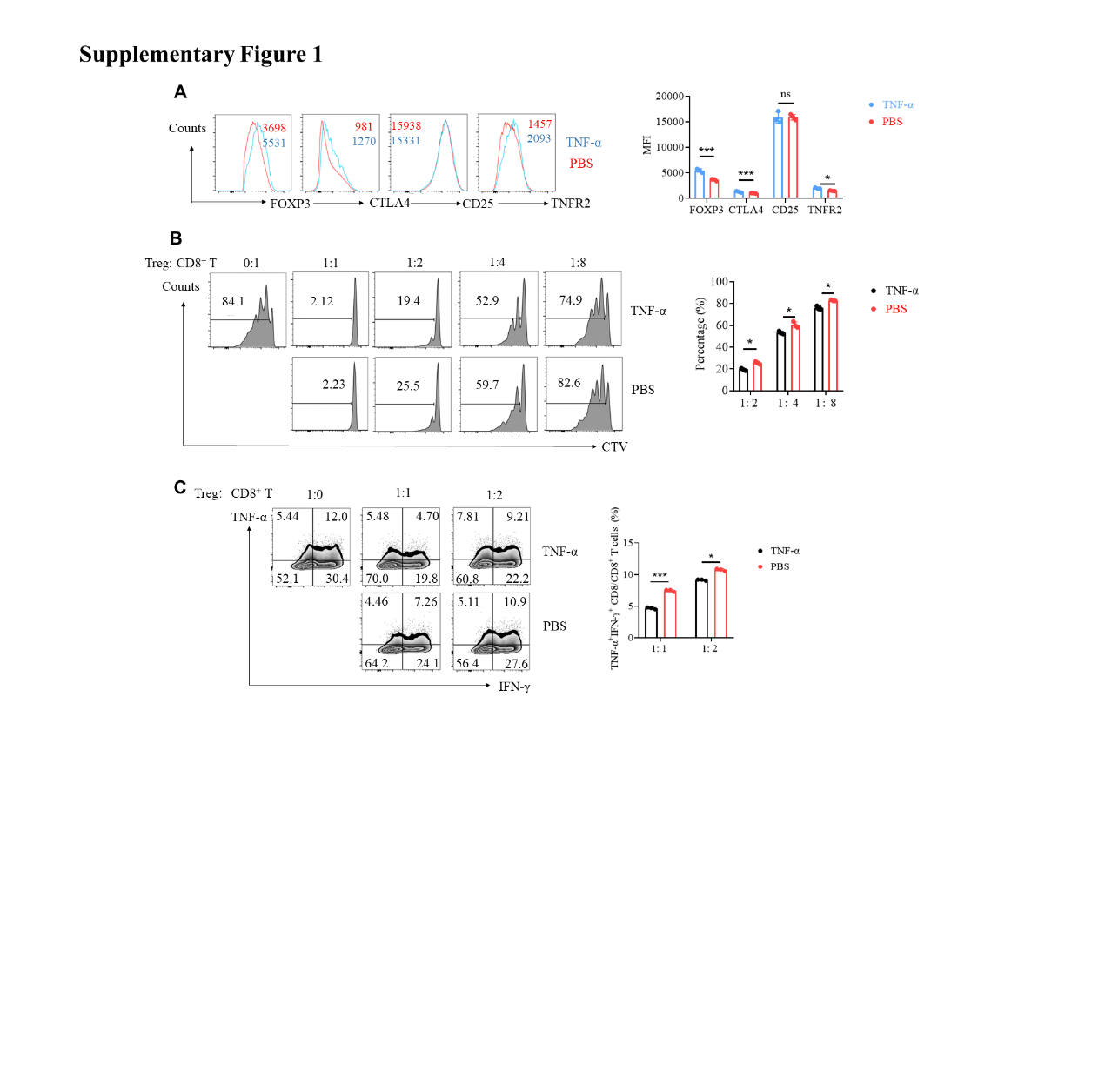
**Supplementary Figure S1.** (A) Tregs derived from healthy human donors were treated with TNF-α (15 ng/ml). Functional markers in Tregs were measured by FACS. (B&C) Tregs derived from healthy human donors were treated with TNF-α (15 ng/ml) or PBS. CD8^+^ T cells were isolated by magnetic beads from healthy donors. Celltrace Violet (CTV) labeled CD8^+^ T cell and Treg treated with or without TNF-α were co-culture for 72 hrs at different ratios (0:1, 1:1, 1:2, 1:4, 1:8). The proliferative ability of CD8^+^ T cells and expression of TNF-α and IFN-γ were measured by FACS. Data are representative of three independent experiments. All data are represented as mean ± sd. *, *P* < 0.05; ***, *P* < 0.001; ns, not significant.


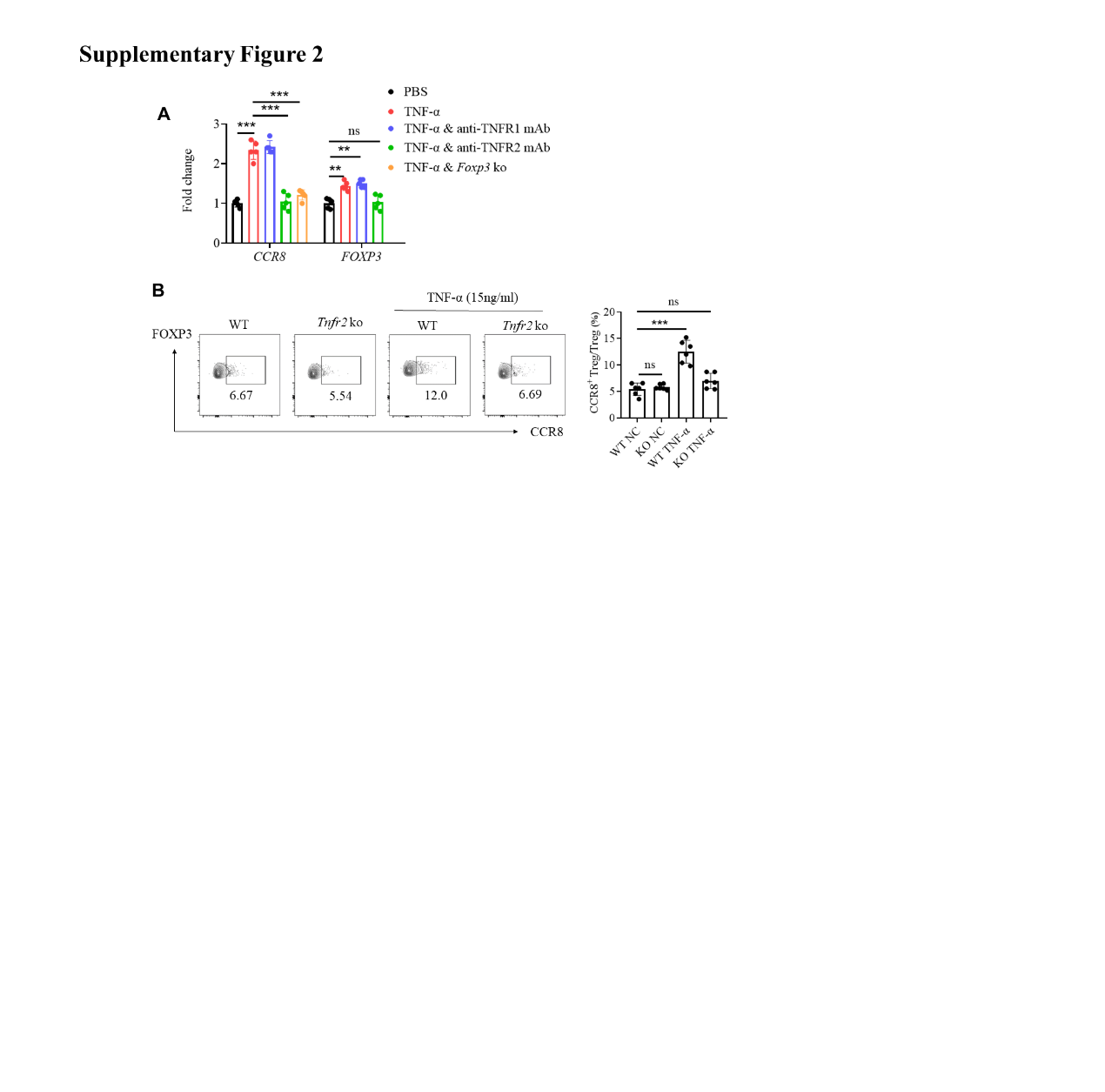


**Supplementary Figure S2.** (A) Tregs derived from healthy human donors were treated with TNF-α (15 ng/ml) and anti-TNFR1 mAb (5 μg/ml) or anti-TNFR2 (5 μg/ml) or *Foxp3* KO condition. The levels of *Ccr8* and *Foxp3* mRNAs were measured by RT-qPCR. (B) Tregs were isolated from *Tnfr2* KO (knockout) and WT (wildtype) mice and Treg were treated with TNF-α (15 ng/ml). Then the percentage of CCR8^+^ Tregs was measured by FACS. **, *P* < 0.01; ***, *P* < 0.001; ns, not significant.


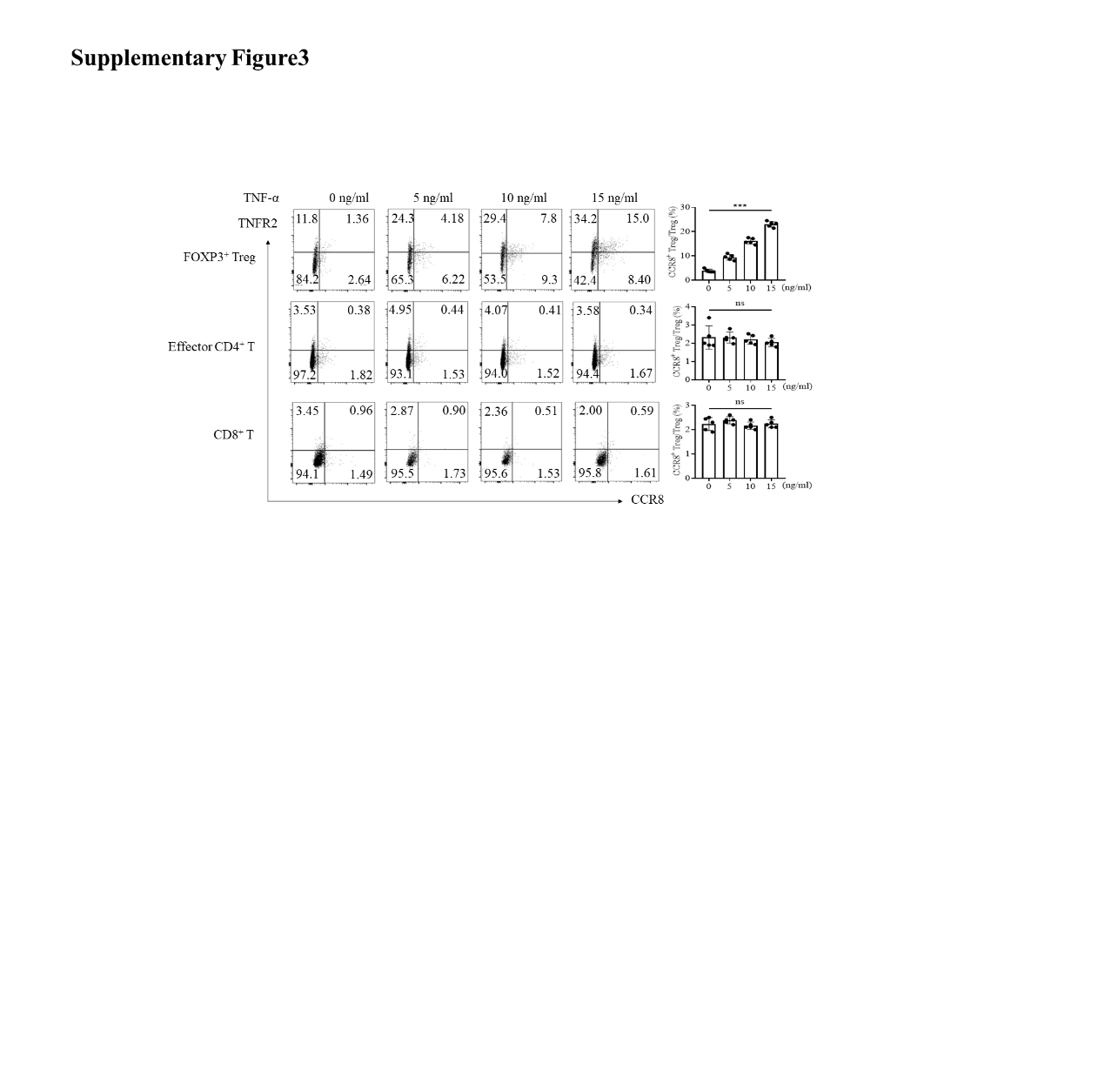
**Supplementary Figure S3.** Treg, CD4^+^ T cells and CD8^+^ T cells were isolated from healthy donors and treated with TNF-α (0, 5, 10, 15 ng/ml). Then the expression of CCR8 and TNFR2 in FOXP3^+^ Tregs, CD4^+^ T effector cells and CD8^+^ T cells were measured by FACS. ***, *P* < 0.001; ns, not significant.


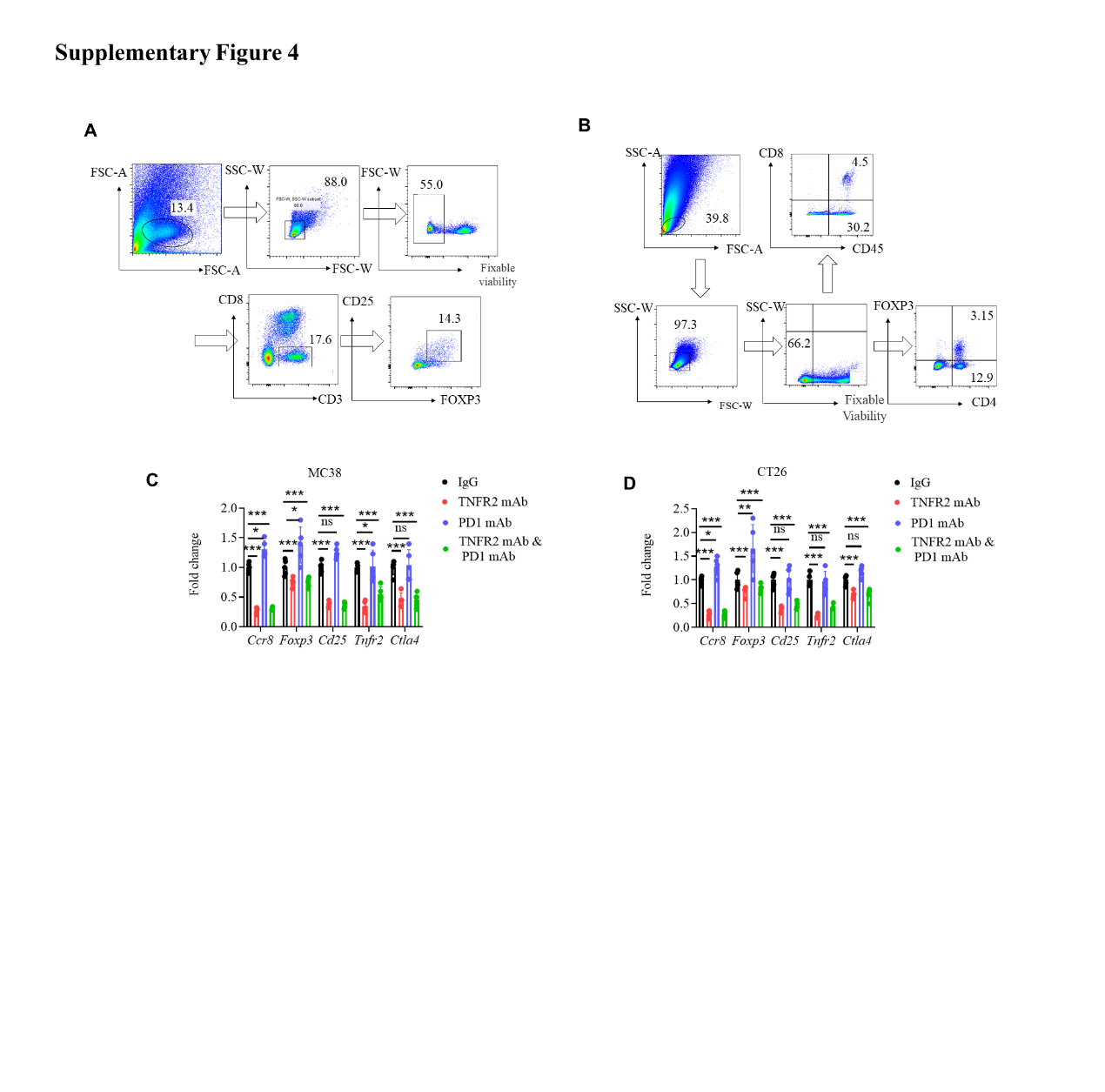
**Supplementary Figure S4.** (A) FACS gating strategies for CD8^+^ T cells and FOXP3^+^ Tregs from human samples. (B) FACS gating strategies for CD8^+^ T cells and FOXP3^+^ Tregs from mouse bearing tumor. (C&D) CD4^+^ T cells were isolated from MC38(C)/ CT26(D) subcutaneous tumors in different treatment groups. The expression levels of *Ccr8, Foxp3*, *Cd25*, *Tnfr2*, and *Ctla4* mRNAs, in CD4^+^ T, were measured by RT-qPCR.
